# Supplementary material for: SegFinder: an automated tool for identifying complete RNA virus genome segments through co-occurrence in multiple sequenced samples
Source: Brief Bioinform. 2025 Jul 24;26(4):bbaf358. doi: 10.1093/bib/bbaf358 (PMC12286774; doi:10.1093/bib/bbaf358)
Supplement: Supplementary_Figures_bbaf358 [file supplementary_figures_bbaf358.pdf]

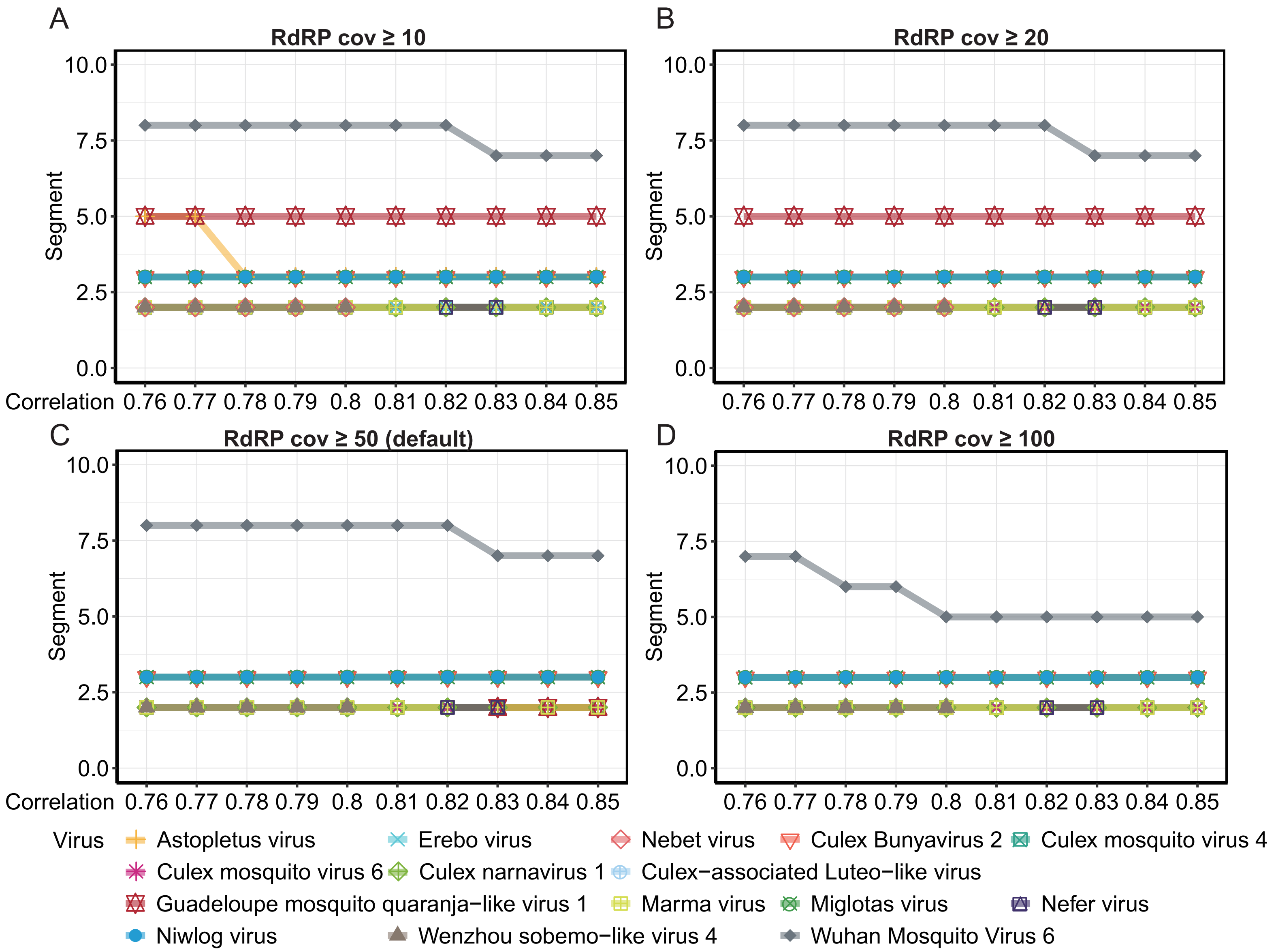

Supplementary Figure 1. Impact of sequencing coverage on the detection of virus segments in the mosquito metatranscriptome data. The number of identified segmented viruses and their segments at RdRP coverage levels of (A) 10, (B) 20, (C) 50, and (D) 100.

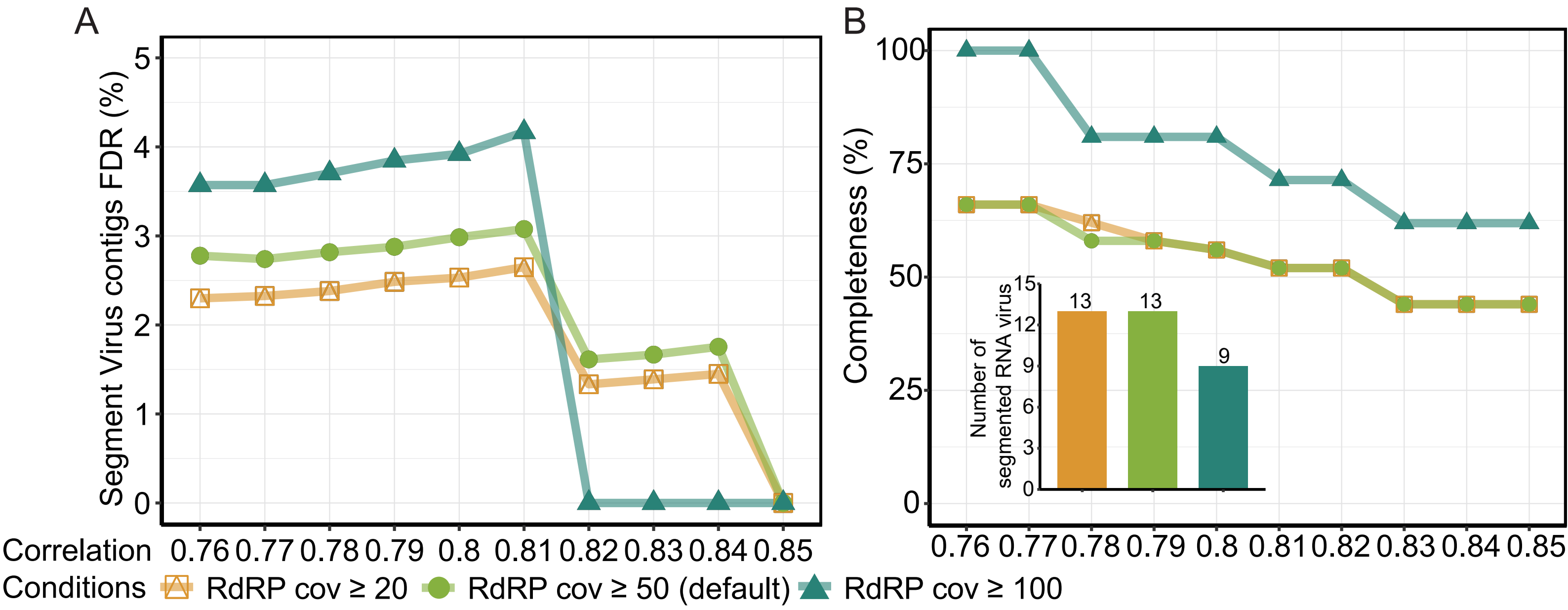

Supplementary Figure 2. Validation of SegFinder parameters across 240 animal and plant metatranscriptomic datasets. (A) False discovery rate (FDR) of predicted viral segments under varying correlation coefficient thresholds and RdRP coverage levels. (B) Segment completeness (%) and number of segmented RNA viruses detected at different correlation thresholds.

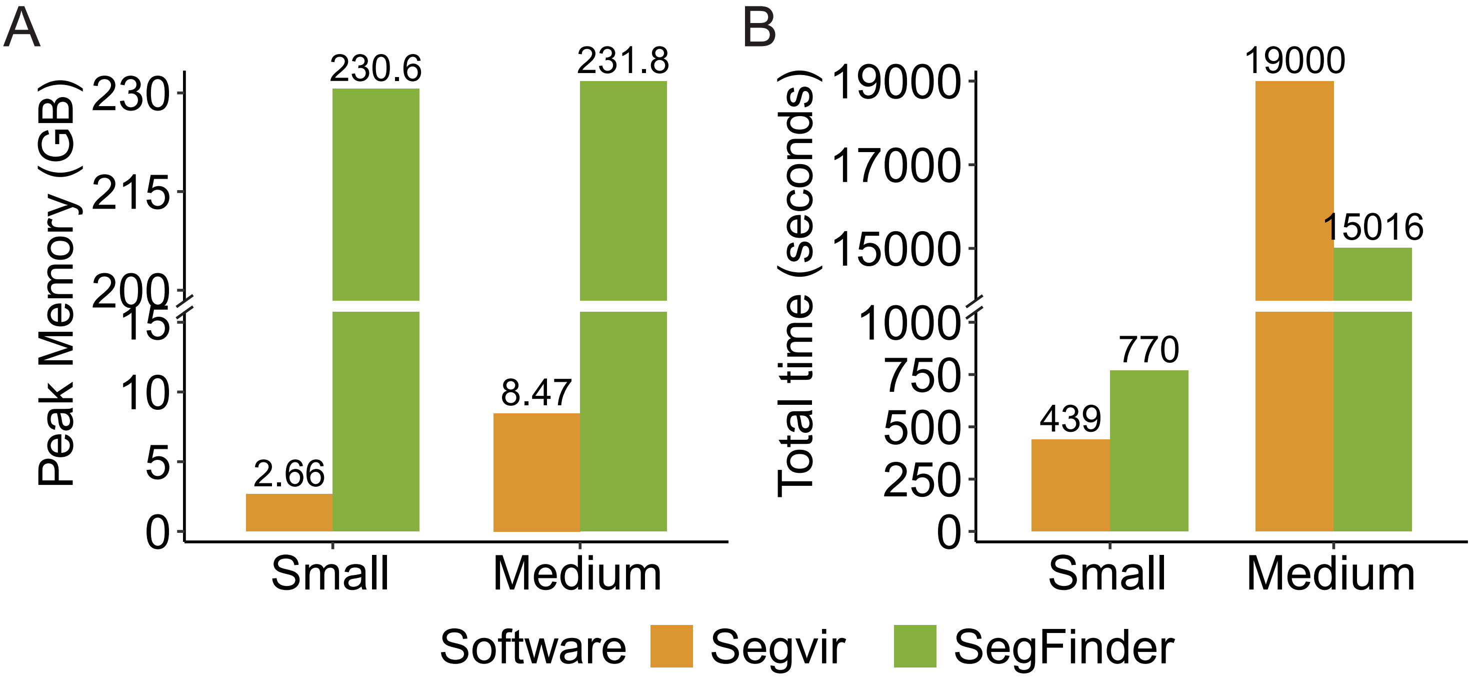

Supplementary Figure 3. Comparison of computational resource usage between SegFinder and SegVir on small and medium-scale datasets. (A) Peak memory consumption (GB) and (B) total runtime (seconds) for processing a small dataset (5 samples, 0.7 Gbp) and a medium dataset (8 samples, 24.58 Gbp).

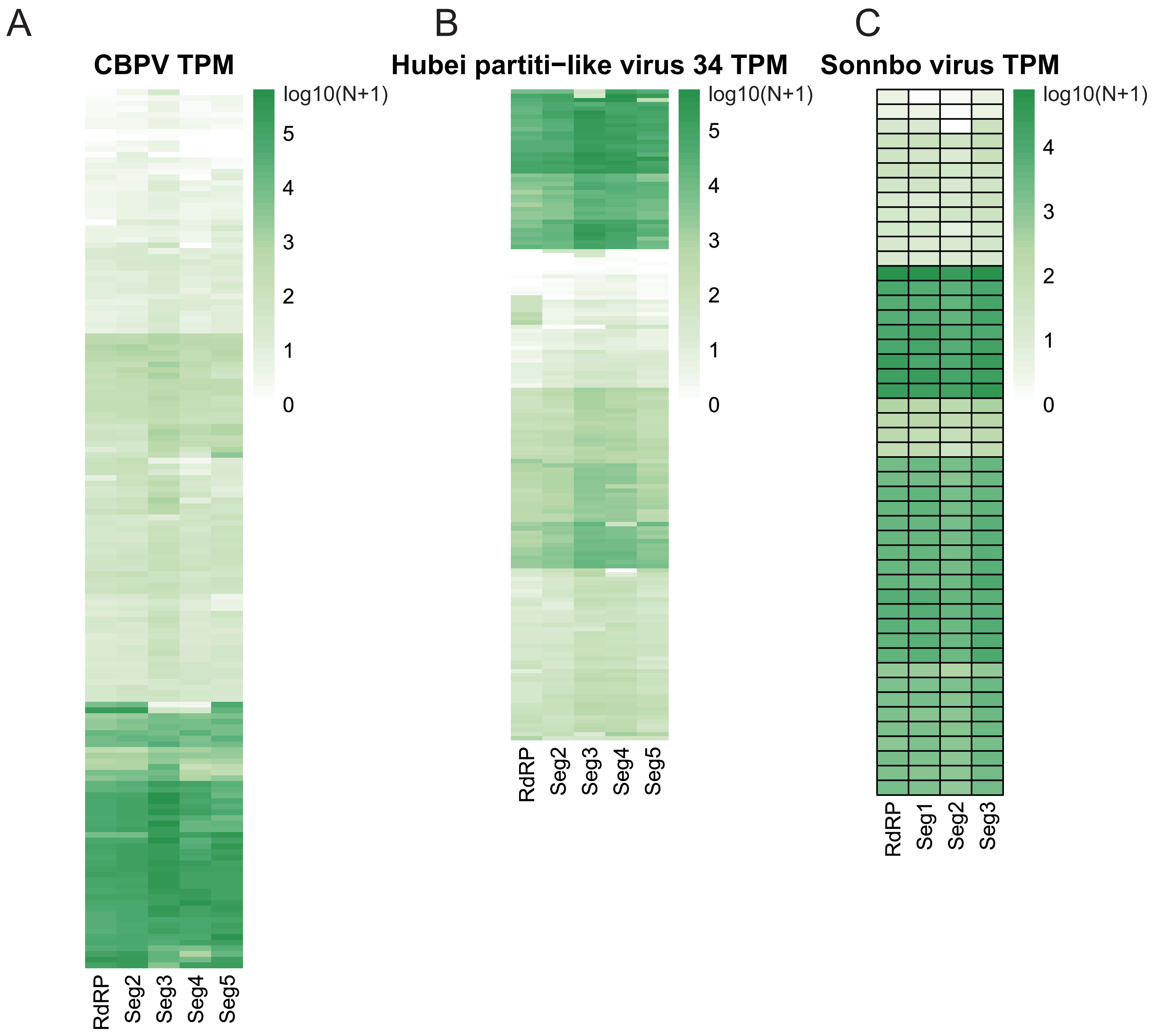

Supplementary Figure 4. TPM validation of segmented viruses of interest. Abundance distribution of (A) chronic bee paralysis virus (CBPV), (B) Hubei partiti-like virus 34, and (C) Sonnbo virus segments in their respective data sets.
