# Supplementary material for: SegFinder: an automated tool for identifying complete RNA virus genome segments through co-occurrence in multiple sequenced samples
Source: Brief Bioinform. 2025 Jul 24;26(4):bbaf358. doi: 10.1093/bib/bbaf358 (PMC12286774; doi:10.1093/bib/bbaf358)
Supplement: Supplementary_table_bbaf358 [file supplementary_table_bbaf358.docx]

Supplementary table 1:

Link: https://figshare.com/ndownloader/files/54298079

Supplementary table 2:

Link: https://figshare.com/ndownloader/files/54298082

Supplementary table 3:

Link: https://figshare.com/ndownloader/files/54298076

Supplementary table 4:

Link: https://figshare.com/ndownloader/files/54298085
