# Supplementary material for: SegFinder: an automated tool for identifying complete RNA virus genome segments through co-occurrence in multiple sequenced samples
Source: Brief Bioinform. 2025 Jul 24;26(4):bbaf358. doi: 10.1093/bib/bbaf358 (PMC12286774; doi:10.1093/bib/bbaf358)
Supplement: Response_to_Briefings_in_Bioinformatics_Editorial_Office_2025_7_2_mang_bbaf358 [file response_to_briefings_in_bioinformatics_editorial_office_2025_7_2_mang_bbaf358.docx]

**Re: Liu et al. BIB-25-0307.R2 “SegFinder: an automated tool for identifying complete RNA virus genome segments through co-occurrence in multiple sequenced samples”**

Dear Editor,

We are delighted to receive the acceptance notification for our manuscript (BIB-25-0307.R2). We sincerely thank you and the reviewers for your valuable guidance and constructive feedback throughout the review process, which has significantly improved the quality of our work.

We have completed the final revisions to our manuscript as requested in your most recent letter. These include: (i) the Biographic Note, (ii) the Key Points section in the required format, and (iii) Alt Text descriptions for all figures. A detailed summary of the changes can be found in the marked-up version of the manuscript. Please let us know if there are any further steps required from our end.

Yours sincerely,

Mang Shi, Professor,

School of Medicine,

Sun Yat-sen University,

Shenzhen, Guangdong, China.

shim23@mail.sysu.edu.cn

**1. Biographical Note:**

We have added the following “Biographical Note” to the title page: “Our team, based at Sun Yan-sen University, uses cutting-edge high-throughput sequencing technology, bioinformatics pipelines, and machine learning algorithm to discover viruses and characterize their interaction with the hosts”.

**2. Key Points Format:**

We have revised the key points section to use the requested bulleted format. The changes have been implemented in the revised manuscript.

**3. Alt Text for Figures:**

We have added comprehensive alt text descriptions for all figures to enhance accessibility:

**Figure 1. SegFinder workflow.** A. Schematic overview of RdRP identification in RNA viruses. The inputs are FASTQ files from multiple meta-transcriptomic libraries. Abbreviations: rRNA (ribosomal RNA), NR (Non-Redundant Protein Sequence Database), NT (Nucleotide Sequence Database). B. The processing pipeline for correlation calculations. Abbreviations: L (Library), C (Contig). C. Schematic illustration of filtering segmented RNA virus clusters. Abbreviations: Cor (Correlation), TPM (Transcripts Per Kilobase Million). **Alt text:** The workflow is illustrated in A–C: A depicts the initial steps, including quality control and viral sequence identification; B follows with abundance estimation for downstream correlation analysis; C represents the final stage, where contig clusters are filtered and identified, serving as evidence for their assignment to the same viral genome.

**Figure 2. Application of SegFinder to individual mosquito metatranscriptomes to optimize parameter settings.** A. False discovery rate for different combinations of SegFinder's coverage parameters compared to known segmented viruses (cov, coverage). B. Line plots showing the completeness of segmented viruses against varying correlation thresholds. C. Results from the application of SegFinder and SegVir to the data set from Batson et al. SegFinder was applied using the optimized parameter settings: a minimum contig length of 600 bp, TPM threshold of 200, RdRP coverage threshold of 50 (10 for other segments), and a correlation coefficient threshold of 0.8. The segmented viruses identified by Batson et al. are represented by slashed squares, those detected by SegFinder are shown as solid squares, and those identified by SegVir are marked with crosshatch symbols. D. Coverage of three genome segments of the newly identified Culex-associated Luteo-like virus. E. Heatmap showing the prevalence and abundance level of Culex-associated Luteo-like virus in each sample. **Alt text:** Finding the best parameter settings for SegFinder, demonstrated in A-D: A shows how false discovery rates vary under different coverage and abundance threshold; B presents segment completeness across a range of correlation thresholds; C highlights the results obtained with the optimal parameters and compares them to those generated by an alternative tool; D-E detail and validate a newly discovered satellite virus.

**Figure 3. Application of SegFinder to vertebrate, invertebrate, plant, and environmental metatranscriptomic data sets.** A. Description of the categories and number of data sets. B. Number of segmented viruses found in all data sets. Blue indicates known segmented viruses; red indicates unknown segmented viruses. C. Percentage of segmented viruses that can be annotated by the nr and nt databases, based on the number of segments. D. Characterization of segmented viruses in various sample types. The colors in the left box represent the superclades to which the segmented viruses belong. Virus segments are represented by blue pentagrams for known segments, red circles for new segments that can be annotated by the nr and nt libraries, and red double circles for new segments that cannot be annotated by the nr and nt libraries. **Alt text:** Application of SegFinder to 858 metatranscriptomes is summarized in A-D: A presents the dataset categories and sample sizes; B estimates the number of novel segmented RNA viruses identified; C highlights that several discovered segments lack detectable homology and are thus difficult to annotate; D displays the complete catalog of identified viral segments.

**Figure 4. Phylogenetic trees illustrating the evolutionary position and segment numbers of the viruses identified in this study.** Phylogenetic trees were estimated at the virus supergroup level using a maximum likelihood method based on analysis of the RdRP protein. The trees are mid-point rooted. Within each tree, existing virus species are marked with blue lines, while newly identified segmented viruses are marked with red lines. The labeling on the right shows the families or genera within each clade, the types of libraries used, and the number of segments found in each segmented virus. Each scale bar represents 0.5 amino acid substitutions per site. **Alt text:** Phylogenetic trees illustrating the evolutionary relationships between segmented RNA viruses identified in this study and previously known viruses, highlighting patterns in segment number evolution.

**Figure 5. Verification of the viral segments detected.** A. The characterization of the segmented viruses and their segments found in the metatranscriptomic libraries. B. TPM values for the eight genome segments of the Wuhan mosquito virus 4 and Wuhan mosquito virus 6 across 48 libraries (represented by vertical green squares). C. Schematic representation of conserved untranslated regions discovered in Beet cryptic virus 1, Beet cryptic virus 2, and Beet partiti-picobirna-like 1. **Alt text:** Confirmation and validation of viral segments are presented in A-D: A summarizes the taxonomic and biological features of each viral segment; B visualizes the segment co-occurrence patterns as supporting evidence for their shared viral origin; C use conserved terminal UTR sequences to further validate that the segments belong to the same virus.
